# Long non‐coding RNA PCAT6 targets miR‐204 to modulate the chemoresistance of colorectal cancer cells to 5‐fluorouracil‐based treatment through HMGA2 signaling

**DOI:** 10.1002/cam4.1809

**Published:** 2019-04-01

**Authors:** Haijun Wu, Qiongyan Zou, Hong He, Yu Liang, Mingjun Lei, Qin Zhou, Dan Fan, Liangfang Shen

**Affiliations:** ^1^ Department of Oncology Xiangya Hospital, Central South University Changsha China; ^2^ Department of Breast and Thyroid The Second Xiangya Hospital, Central South University Changsha China; ^3^ Department of Medical Records Information The First Hospital of Changsha Changsha China

**Keywords:** 5‐fluorouracil, chemoresistance, colorectal cancer, HMGA2, lncRNA prostate cancer‐associated transcript 6, miR‐204

## Abstract

Colorectal cancer (CRC) is still the third most common cancer in the world with a limited prognosis due to the chemoresistance of CRC cells to 5‐fluorouracil (5‐FU)‐based chemotherapy. In our previous study, we revealed that miR‐204 overexpression could sensitize CRC cell to 5‐FU treatment through targeting HMGA2/PI3K signaling pathway; however, miR‐204 expression in CRC tissues is abnormally downregulated. Long non‐coding RNAs (lncRNAs) dysregulation has been reported in human diseases, including cancer. Also, lncRNA can regulate cancer cell proliferation, invasion, migration, as well as chemoresistance. LncRNA prostate cancer‐associated transcript 6 (PCAT6) acts as an oncogene in many cancers; herein, PCAT6 expression was abnormally upregulated in CRC tissues and cell lines, suggesting its potential role in CRC. Further, we assessed the specific function and mechanism of PCAT6 in CRC. Furthermore, we revealed that PCAT6 knockdown attenuated CRC chemoresistance to 5‐FU through miR‐204/HMGA2/PI3K; miR‐204 inhibition could partially reverse the effect of PCAT6 knockdown. Taken together, we demonstrate that the abnormal PCAT6 overexpression inhibits miR‐204 expression in CRC, thereby promoting HMGA2/PI3K signaling activity, ultimately enhancing the chemoresistance of CRC cells to 5‐FU; PCAT6 represents a promising target for dealing with CRC chemoresistance.

## 1 INTRODUCTION

Colorectal cancer (CRC) is a common malignancy in the digestive system.^1^ Due to late diagnosis and the acquisition of chemoresistance to first‐line chemo agents, including 5‐fluorouracil (5‐FU),^2^ oxaliplatin,^3^ and raltitrexed,^4^ the prognosis of patients with CRC is still poor, and frequently accompanied by cancer recurrence or metastasis shortly after surgery treatment. In our previous study, we revealed that miR‐204 overexpression could sensitize CRC cell to 5‐FU‐based therapy via targeting HMGA2/PI3K signaling;^5^ however, the miR‐204 expression is abnormally downregulated in CRC tissues. Further investigating the mechanism of miR‐204 dysregulation in CRC may provide a novel angle of ameliorating the chemoresistance of CRC to 5‐FU treatment.

Long non‐coding RNAs (lncRNAs) are transcripts with a length of >200 nt; due to their inability of encoding proteins, they were initially considered as transcriptional “noise”.^6^ However, recently, lncRNAs have been regarded as crucial regulators in almost all aspects of biology,^7^, ^8^, ^9^ including metabolism,^10^ cellular development,^11^ as well as disease progression,^12^, ^13^, ^14^, ^15^ rather than nonsense fragments. The mechanisms by which lncRNAs exert their effects may be closely related with the secondary or tertiary structures of lncRNAs,^16^ such as acting as molecular scaffolds,^17^ aiding alternative splicing^18^ in the nucleus or affecting translation, increasing or suppressing the degradation of downstream mRNAs, and serving as miRNA sponges in cytoplasm.^19^, ^20^, ^21^, ^22^

In recent decades, studies have focused on dysregulation of lncRNAs participates in pathophysiological processes of human diseases, including cancer.^23^ Some lncRNAs have primarily been found to be associated with tumor invasion, metastasis, and even multi‐drug resistance.^19^, ^24^, ^25^ For example, lncRNA H19 works as an miRNA precursor of miR‐675 and an endogenous miRNA sponge of let‐7 to affect CRC cell proliferation and EMT.^26^ LncRNA prostate cancer‐associated transcript 6 (PCAT6) (also known as PCAN‐R1, ncRNA‐a2, and KDM5B‐AS1) was first identified in keratinocytes and indirectly activates the Wnt/β‐catenin pathway by interacting with KLHL12 in cervical cancer cells.^27^ With the use of lncRNA microarray, PCAT6 was further confirmed as the most upregulated lncRNA in cancer tissues and significantly correlated with the metastasis of prostate cancer.^28^ In lung cancer, PCAT6 was also found to be upregulated using Affymetrix HG‐U133 Plus 2.0 Array with a lncRNA classification pipeline.^29^ However, the role of PCAT6 in CRC has not been reported. Interestingly, PCAT6 has been predicted by online tools to possess a possible binding site of miR‐204.

Herein, we assessed the specific functions of PCAT6 in CRC through measurement of cell viability, DNA synthesis ability, as well as the chemoresistance of cancer cells to 5‐FU‐based therapy in response to PCAT6 knockdown. Also, we validated the interaction between PCAT6 and miR‐204, and the potential mechanism. Finally, we investigated whether PCAT6 could affect the chemoresistance of CRC cell to 5‐FU‐based therapy through miR‐204‐mediated HMGA2/PI3K signaling pathway. Taken together, we provided a new angle of ameliorating CRC chemoresistance to 5‐FU‐based chemotherapy.

## 2 MATERIALS AND METHODS

### 2.1 Cell lines, tissues, and transfection

Seventy‐three paired CRC tissues, and the adjacent non‐cancerous tissues were collected from patients who underwent surgical resection at Xiangya Hospital, Central South University under the approval of the Ethics Committee of Xiangya Hospital, Central South University (Changsha, China). Informed consent was signed by all patients enrolled. Tissue specimens were later stored in liquid nitrogen for further RNA extraction or fixed in 4% paraformaldehyde 24 hours for pathological grading. The clinical parameters of cased enrolled are listed in Table 1, and analysis results by COX regression model are shown in Table 2.

**Table 1:** Correlation between lncRNA PCAT6 expression and clinicopathological features

Characteristics	PCAT6 expression		*P*‐value
	High	Low	
Age			
<50	18	14	0.401
≥50	19	22	
Gender			
Female	13	13	0.931
Male	24	23	
Tumor size			
<5	12	22	0.014
≥5	25	14	
Tumor location			
Colon	15	13	0.697
Rectum	22	23	
TNM stage			
Ⅰ	2	7	0.007
Ⅱ	10	19	
Ⅲ	19	7	
Ⅳ	6	3	
Lymph node metastasis			
No	8	16	0.038
Yes	29	20	
Differentiation			
Moderate‐Poor	23	18	0.295
Well	14	18	

M, describes distant metastasis; N, describes adjacent lymph nodes that are involved; TNM, T describes the size of the original tumor and whether it has invaded nearby tissue.

**Table 2:** Univariate and multivariate analysis for factors related to overall survival using the Cox proportional hazard model

Characteristics	Univariate analysis			Multivariate analysis		
	*P*	HR	95%CI	*P*	HR	95% CI
Age						
<50 vs ≥50	0.260	1.449	0.760‐2.762	N.A		
Gender						
Female vs Male	0.686	1.147	0.589‐2.234	N.A		
Tumor size						
<5 vs ≥5	0.080	0.551	0.283‐1.074	0.883	0.940	0.409‐2.157
Tumor location						
Colon vs Rectum	0.455	0.769		N.A		
TNM stage						
Moderate‐Poor vs Well	0.014			0.030		
Ⅰ	0.007	0.160	0.042‐0.604	0.047	0.204	0.042‐0.982
Ⅱ	0.007	0.299	0.125‐0.715	0.021	0.309	0.114‐0.839
Ⅲ	0.019	0.340	0.138‐0.837	0.005	0.246	0.093‐0.651
Ⅳ				N.A		
Lymph node metastasis						
No vs Yes	0.152	0.587	0.283‐1.217	N.A		
Differentiation						
Moderate‐Poor vs Well	0.801	0.920	0.482‐1.755	N.A		
PCAT6						
High vs Low	0.039	2.020	1.035‐3.940	0.038	2.197	1.044‐4.625

#### 2.1.1 Information for cell lines and cell transfection

Seven CRC cell lines: HCT116, HT‐29, SW620, SW480, DLD‐1, RKO, and LoVo, a normal colon fibroblast cell line CCD‐112CoN and a kidney epithelial cell line, HEK293 were all obtained from the American Type Culture Collection (Cat:CCL‐247, HTB‐38, CCL‐227, CCL‐211, CRL‐2577, CLL‐229, CRL‐1541, CRL‐1573, ATCC, Manassas, VA, USA) and cultured in RPMI‐1640 medium (Cat. 21875091, Gibco, Waltham, MA, USA) with 10% FBS (Cat. 10091148; Gibco) at 37 °C with 5% CO_2_.

Si‐NC or si‐PCAT6 (Cat. A09001; Genepharma, Shanghai, China) transfection was performed to achieve PCAT6 knockdown by using Lipofectamine 2000 (Cat. 11668019; Invitrogen, Waltham, MA, USA). MiR‐204 mimics or miR‐204 inhibitor (Cat. B01001 and B03001; Genepharma) transfection was performed to achieve miR‐204 overexpression or miR‐204 inhibition. The sequences of siRNA and miRNA mimics or inhibitor were shown in Table S1.

### 2.2 Quantitative real‐time PCR

Trizol reagent (Cat. 15596018; Invitrogen) was used to extract the total RNA from cells and tissue sample following the manufacturer's protocols. One microgram total RNA was reverse transcribed using miRNA‐specific primer and the miScript Reverse Transcription kit and miScript SYBR Green PCR kit (Cat.218161 and 219073; Qiagen, Hilden, Germany) was used for miRNA qRT‐PCR using U6 as an endogenous control. The annealing temperature is 60°C. The First‐strand cDNA synthesis kit and Power SYBR™ Green PCR Master Mix (Cat. K1612 Invitrogen, Cat. 4368577, ABI, San Jose, CA, USA) were used for the reverse transcription and qRT‐PCR for measurement of PCAT6 and HMGA2 expression using GAPDH mRNA as an endogenous control. The above QRT‐PCR assays were conducted using ABI 7900HT Real‐time PCR System (Applied Biosystems, Waltham, MA, USA) The 2^−ΔΔCt^ method was used for data processing.^30^ The primers were shown in Table S1.

### 2.3 Immunoblotting

The protein levels of HMGA2, PI3K, p‐PI3K, p‐Akt, Akt in CRC cells were measured using immunoblotting assays. Cells were lysed in RIPA lysis buffer (Cat. P0013B, Beyotime, Shanghai, China), and the protein concentration was evaluated by BCA Protein Assay Kit (Cat. P0010, Beyotime Tech.). The 20‐50 μg of protein samples were loaded onto 10% SDS‐PAGE minigel and then transferred onto a 0.2 μm PVDF membrane (Cat. 1620177, Bio‐Rad, Hercules, CA, USA) by Bio‐Rad Mini Trans‐Blot® systems (Bio‐Rad). Afterward, the membrane was probed with the antibodies listed below: anti‐HMGA2 (ab97276, Abcam, Cambridge, UK), anti‐PI3K (ab86714, Abcam), anti‐p‐PI3K (ab182651, phospho Y607, Abcam), anti‐Akt (ab32505, Abcam), anti‐p‐Akt (ab81283, phospho S473, Abcam), and anti‐β‐actin (ab6276, Abcam; 1:1000 in TBST) at 4°C overnight, and incubated with HRP‐conjugated secondary antibody for 1 hour at room temperature (1:5000 in TBST, Cat. sc‐2004 and sc‐2005, Santa Cruz, Co. Ltd, Sant Cruz, CA, USA). Signals were visualized using ECL Substrates (Cat. 345818, Millipore, Billerica, MA, USA) and exposed to X‐film (Cat. X‐OMAT, Kodak, Rochester, NY, USA). The immunoblots were quantified using imageJ software (NIH, National Institute of Health, Bethesda, MD, USA) and normalized to endogenous β‐actin. The original blots were shown in Figure S1.

### 2.4 Luciferase activity

The fragments of wild‐type PCAT6 and mutant PCAT6 (containing a 5‐bp mutation in the predicted binding site of miR‐204) were cloned into the pGEM‐T easy vector system (wt‐PCAT6, mut‐PCAT6; Cat. A1260, Promega, Madison, WI, USA). The primers were shown in Table S1. HEK293 cells were seeded into a 24‐well plate and cultured overnight. The 0.5 μg of pGL4 luciferase expression vector (Cat. E6651, Promega), 0.5 μg wt‐PCAT6 or mut‐PCAT6 plasmid and 50 nM miR‐204 mimics or miR‐204 inhibitor (Cat.B03001, B02003, GenePharma) were cotransfected into HEK293 cells using 3 μL lipofectamine2000 (Invitrogen). After 48 hours transfection, Dual‐Luciferase Reporter Assay System (Cat. E1910, Promega) was used to perform the luciferase assays. The ratio of Firefly/Renilla luciferase activity was determined by GloMax® Discover Multimode Microplate Reader (Promega).

### 2.5 MTT assay

Cells were seeded into 96‐well plates (5000 cells per well). Twenty‐four hours later, cells were transfected with si‐PCAT6. Twenty‐four hours later, cells were treated with 5‐FU (1, 2, 4, 8, 16, 32, 64 μg/mL, Cat. F6627, Sigma‐Aldrich, St. Louis, MO, USA) for 24 hours. Then, 20 μL MTT (5 mg/mL; Cat. M2003, Sigma‐Aldrich) was added in the culture medium, and the cells were incubated for an additional 4 hours. Finally, after discarding the supernatant, 200 μL DMSO (Cat. TS‐20688, Thermo Fisher, Waltham, MA, USA) was added to dissolve the formazan. The optical density was examined at 490 nm using a plate reader (ELx808 Bio‐Tek Instruments, Hercules, CA, USA). The relative cell viability was calculated using the non‐treated cells (control) as 100%; the 5‐FU concentration to reduce cell viability to 50% was defined as IC50 values.

### 2.6 BrdU incorporation assay

DNA synthesis capacity was determined using BrdU assays according to the methods reported previously.^31^ Briefly, cells were plated in 96‐well plates (2 × 10^3^ cells/well), cultured for 24 or 48 hours and incubated with a final concentration of 10 μM BrdU (Cat. 550891, BD Pharmingen, San Jose, CA, USA) for 2 hours. Then, the cells were fixed with 4% polyoxymethylene for 30 minutes and permeated with 0.3% Triton X‐100 for 10 min at room temperature (Cat. P0099 and P0096, Beyotime). And then cells incubated with peroxidase‐coupled anti‐BrdU‐antibody (Cat. B8434, Sigma‐Aldrich) for 1 hour, washed three times with PBS, incubated with peroxidase substrate (tetramethylbenzidine, Cat.860336; Sigma‐Aldrich) for 30 minutes, and the 450 nm absorbance values were measured for each well. BrdU absorbance in cells treated with BrdU antibody alone was taken as the background value.

### 2.7 Statistics analysis

Data were processed using SPSS 17.0 statistical software (SPSS, Armonk, NY, USA) and presented as mean ± SD of three biological replicates. All data were analyzed by normality test first using Kolmogorov‐Smirnov test. Significance was analyzed using a two‐tailed unpaired Student's *t*‐test. Paired Student's *t*‐test was used to compare the expression of miR‐204 and PCAT6 in colorectal cancer tissues and normal tissues. Chi‐square test was used for analyzing the correlation of the expression of PCAT6 with clinicopathologic features, Cox regression model was used for univariate and multivariate analysis for factors related to overall survival, log‐rank test was used for analyzing overall survival; Spearman's rank correlation analysis was used for analyzing correlation in PCAT6, miR‐204, and HMGA2. *P* values of <0.05 were considered statistically significant.

## 3 RESULTS

### 3.1 The expression of PCAT6 in CRC tissues and its correlation with clinicopathological features in patients with CRC

**Figure 1:**
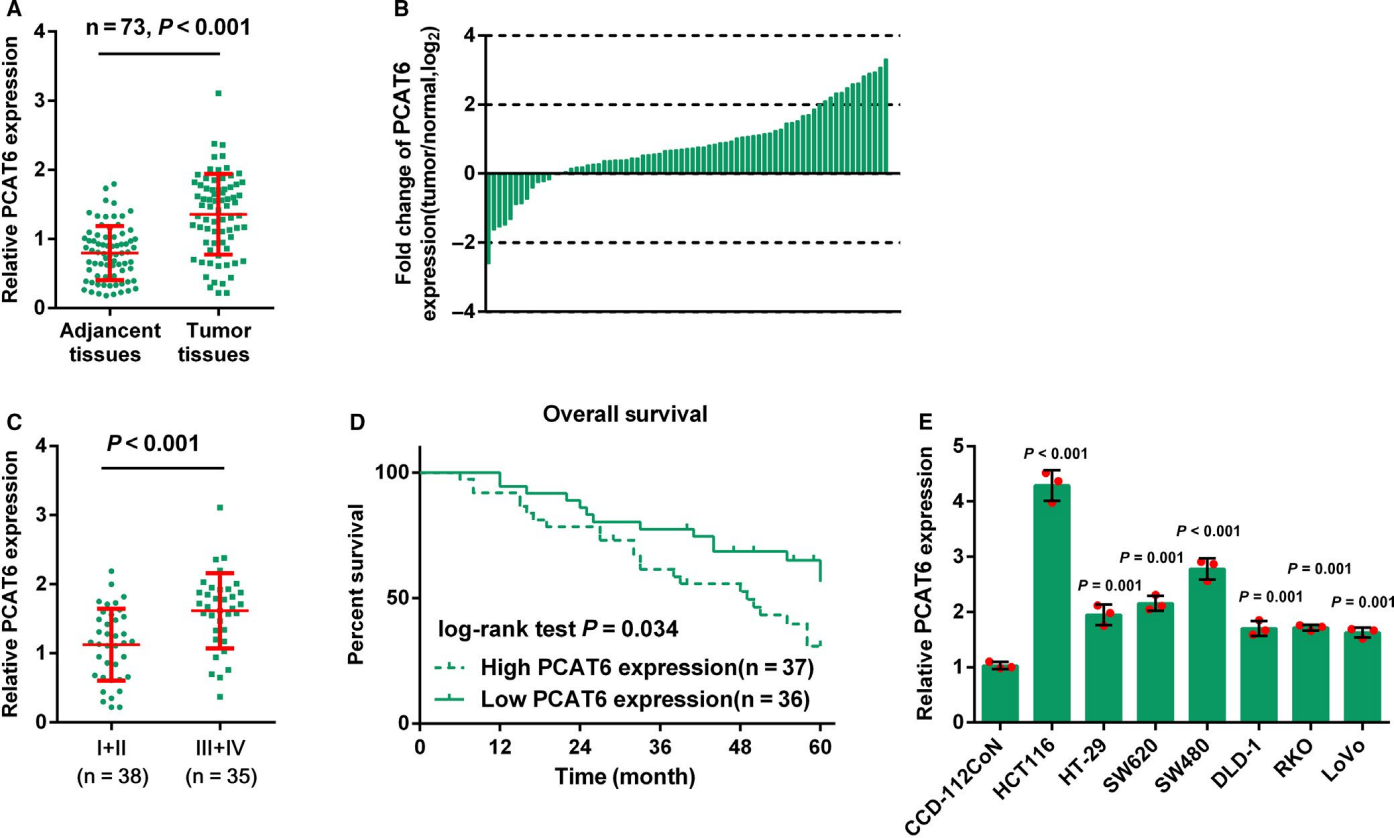
The expression of PCAT6 in CRC tissues and its correlation with clinicopathological features in patients with CRC. (A) The expression of PCAT6 in 73 paired CRC and adjacent normal tissues was determined using real‐time PCR assays. (B) Fold changes of PCAT6 expression was exhibited as log_2_ (tumor/normal). (C) Expression of PCAT6 in CRC tissues grouped by TNM stages (I + II vs III + IV), compared with matched adjacent normal tissues. (D) Kaplan‐Meier overall survival curves for 73 patients with CRC classified according to relative PCAT6 expression level. (E) The expression levels of PCAT6 in seven CRC cell lines, HCT116, HT‐29, SW620, SW480, DLD‐1, RKO, and LoVo, and a normal colon fibroblast cell line, CCD‐112CoN, were determined using real‐time PCR. The data are presented as mean ± SD of three independent experiments

To investigate the detailed function of PCAT6 in CRC, we examined PCAT6 expression in CRC tissues. In 73 CRC specimens, PCAT6 expression was significantly up‐regulated, compared to that in adjacent normal tissues (Figure 1A,B). Also, PCAT6 expression was much higher in samples of advanced TNM stages (III + IV) than that in early stage (I + II; Figure 1C). A total of 73 CRC specimens were grouped according to the PCAT6 expression: a high PCAT6 expression group (samples possessing PCAT6 expression higher than the median value, n = 37) and a low PCAT6 expression group (samples possessing PCAT6 expression lower than the median value, n = 36; Table 1). According to clinicopathological feature analysis using chi‐square test, higher PCAT6 expression was correlated with larger tumor size (*P* = 0.014), advanced TNM stages (*P* = 0.007), and lymph node metastasis (*P* = 0.038). The overall survival time and pathologic features of 73 patients were analyzed using the COX risk proportional regression model. As shown in Table 2, univariate analysis revealed that PCAT6 expression and TNM stage caused significant differences in overall survival in patients with CRC; multivariate analysis revealed that high PCAT6 expression was of high risk (HR = 2.197, 95% CI = 1.044‐4.625). The overall survival in patients with CRC obtaining a higher PCAT6 expression was shorter than that obtaining a lower PCAT6 expression (*P* = 0.034, Figure 1D). To further investigate the effect and the mechanism of PCAT6 in CRC, the expression levels of PCAT6 in seven CRC cell lines, HCT116, HT‐29, SW620, SW480, DLD‐1, RKO, and LoVo, as well as a normal colon fibroblast, CCD‐112CoN cells were determined by real‐time PCR assays. PCAT6 expression was remarkably increased in the above CRC cells, compared to that in CCD‐112CoN cells (Figure 1E). Moreover, PCAT6 expression was much higher in HCT116 and SW480 cells; therefore, these two cell lines were selected as cell models for further experiments.

### 3.2 The effect of PCAT6 on CRC cell proliferation and chemoresistance to 5‐FU

**Figure 2:**
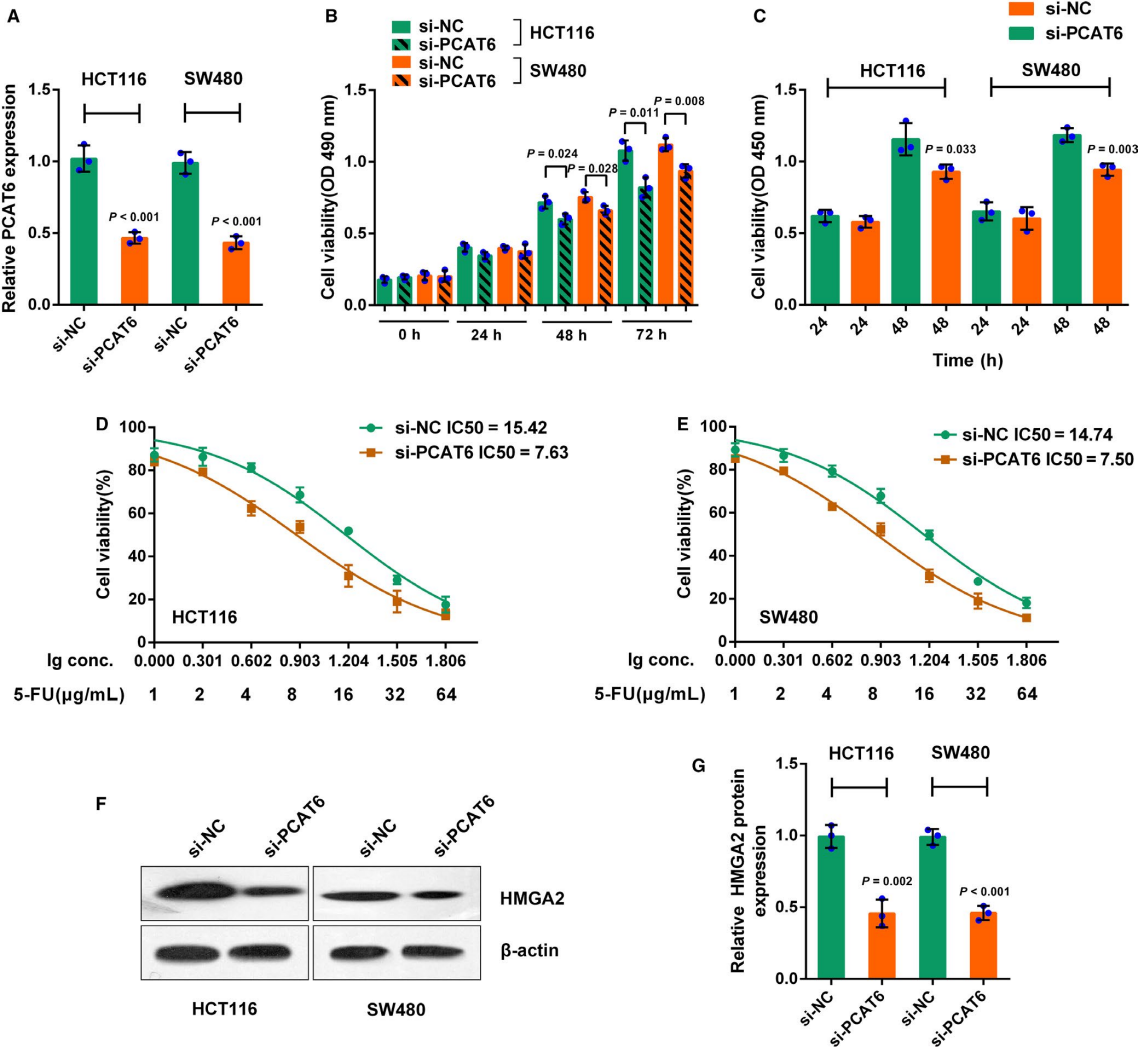
The effect of PCAT6 on CRC cell proliferation and chemoresistance to 5‐FU. (A) HCT116 and SW480 cells were transfected with si‐PCAT6 to achieve PCAT6 knockdown, as confirmed real‐time PCR assays. (B) The cell viability was determined using MTT assays. (C) The DNA synthesis ability was assessed using BrdU assays. (D‐E) HCT116 and SW480 cells were treated with a series of doses of 5‐FU (1, 2, 4, 8, 16, 32, 64 μg/mL) for 24 h; the cell viability was monitored using MTT assays. Data were displayed as a percentage normalized to the cell viability with no 5‐FU treatment. The abscissa was the logarithm of 5‐FU concentration (log‐conc.). LC50 represented the concentration of 5‐FU when cell viability was reduced to 50%. (F‐G) The protein levels of HMGA2 were determined using Western blot assays. The data are presented as mean ± SD of three independent experiments

In order to evaluate the specific role of PCAT6 in CRC, PCAT6 siRNA was transfected into HCT116 and SW480 cell lines to conduct PCAT6 expression, as confirmed by real‐time PCR assays (Figure 2A). The cell viability and DNA synthesis ability of HCT116 and SW480 cells in response to PCAT6 knockdown were determined by MTT and BrdU assays, respectively. PCAT6 knockdown remarkably suppressed CRC cell proliferation (Figure 2B,C). HCT116 and SW480 cell lines were subjected to different doses of 5‐FU (1, 2, 4, 8, 16, 32, and 64 μg/mL) for 24 hours; next, MTT assays were performed for the cell viability. The results showed that 5‐FU suppressed the cell viability in a concentration‐dependent manner; after the PCAT6 knockdown, the IC50 values of HCT116 and SW480 cells were reduced from 15.42 and 14.74 to 7.63 and 7.50, respectively (Figure 2D,E). In our previous study, the HMGA2/PI3K signaling pathway is involved in CRC cell proliferation;^5^ herein, we investigated whether PCAT6 could regulate HMGA2. In HCT116 and SW480 cells, PCAT6 knockdown significantly reduced the protein levels of HMGA2 (Figure 2F,G). The data suggest that PCAT6 may affect CRC chemoresistance to 5‐FU, involving HMGA2 signaling.

### 3.3 PCAT6 directly binds to miR‐204 to inhibit its expression

**Figure 3:**
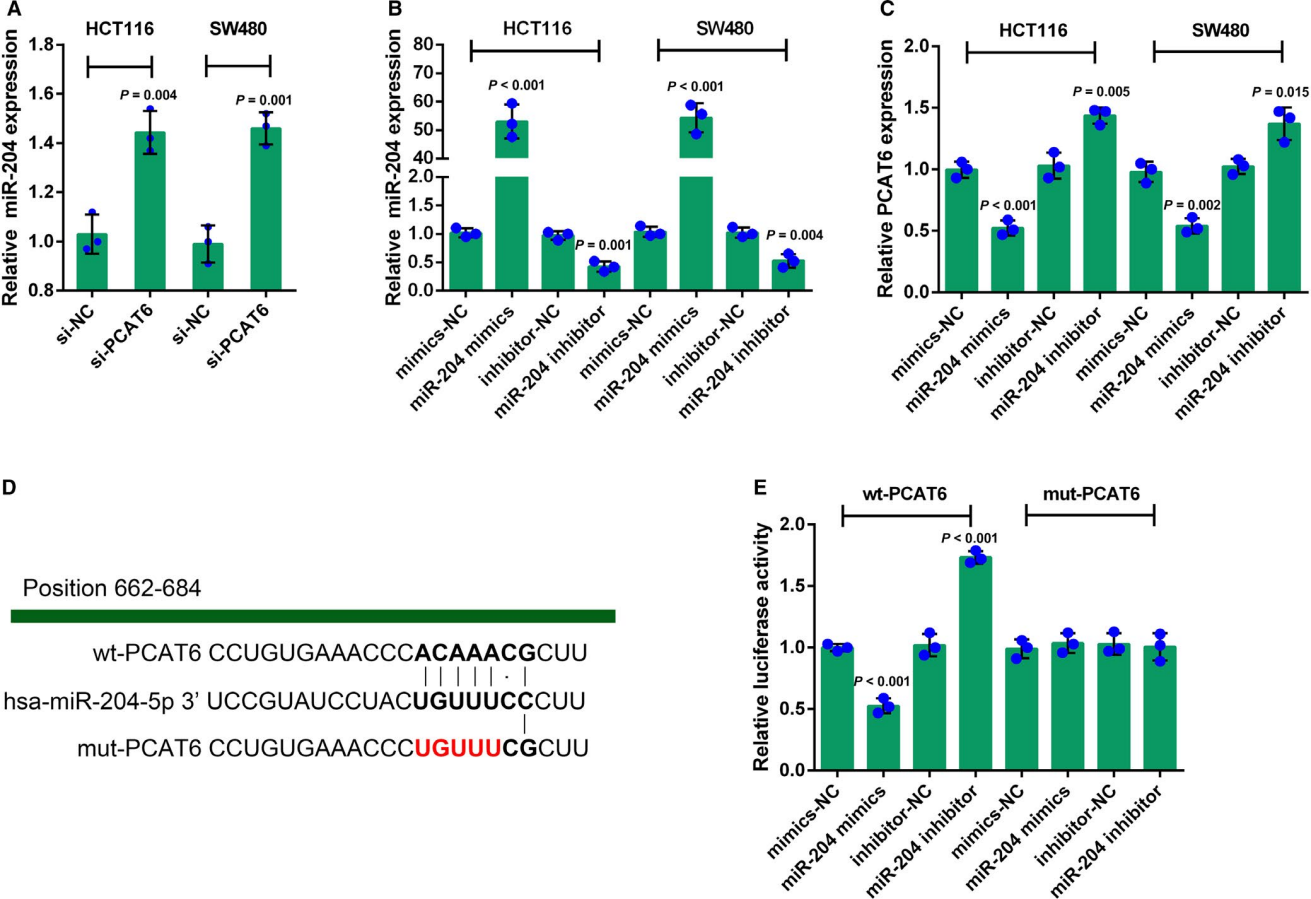
PCAT6 directly binds to miR‐204 to inhibit its expression (A) HCT116 and SW480 cells were transfected with si‐PCAT6; the expression of miR‐204 was determined using real‐time PCR assays. (B) HCT116 and SW480 cells were transfected with miR‐204 mimics or miR‐204 inhibitor to achieve miR‐204 overexpression or miR‐204 inhibition, as confirmed using real‐time PCR assays. (C) PCAT6 expression in response to miR‐204 overexpression or miR‐204 inhibition was determined using real‐time PCR assays. (D) A wild‐type and a mutant‐type luciferase reporter gene vector were constructed by cloning the PCAT6 fragment into the downstream of the Renilla psiCHECK^TM^‐2 vector, named wt‐PCAT6 and mut‐PCAT6, respectively. In the mut‐PCAT6 vector, the seed region of the PCAT6 was mutated to remove all complementarity to nucleotides 2‐5 of miR‐204. (E) The above vectors were cotransfected into HEK293 cells with miR‐204 mimics or miR‐204 inhibitor, respectively; the luciferase activity was determined using the Dual‐Luciferase Reporter Assay System. The data are presented as mean ± SD of three independent experiments

Non‐coding RNAs (ncRNAs), could exert an extensive series of biological effects through forming interaction network,^32^ such as miRNA‐miRNA synergistic network.^33^ Moreover, lncRNA has been reported to target miRNAs, thus interacting with miRNAs and participating in either normal physiological processes or pathogenic processes.^34^ We revealed that miR‐204/HMGA2 axis modulates CRC cell sensitivity to 5‐FU;^5^ herein, we investigated whether PCAT6 could interact with miR‐204, thereby affecting CRC chemoresistance to 5‐FU. The expression of miR‐204 was determined in si‐PCAT6 transfected HCT116 and SW480 cells by real‐time PCR assays. PCAT6 knockdown remarkably increased miR‐204 expression in the above two CRC cell lines (Figure 3A). In order to evaluate the effect of miR‐204 on PCAT6 expression, these two CRC cell lines were transfected with miR‐204 mimics or miR‐204 inhibitor, as confirmed by real‐time PCR assays (Figure 3B); as revealed by real‐time PCR assays, PCAT6 expression was negatively regulated by miR‐204 in CRC cells (Figure 3C). Furthermore, we performed luciferase reporter gene assay to validate the mechanism of PCAT6 interacting with miR‐204. Wild‐type and mutant‐type luciferase reporter gene vectors were constructed by cloning the PCAT6 fragment into the downstream of the Renilla psiCHECK**^TM^**‐2 vector, named wt‐PCAT6 and mut‐PCAT6, respectively. To generate the mut‐PCAT6 vector, the seed region of the PCAT6 was mutated to remove all complementarity to nucleotides 2‐5 of miR‐204 (Figure 3D). HEK293 cells were cotransfected with these vectors and miR‐204 mimics or miR‐204 inhibitor, respectively, and then subjected to luciferase activity determination. The luciferase activity of wt‐PCAT6 was significantly suppressed by miR‐204 overexpression whereas enhanced by miR‐204 inhibition; after mutating the possible miR‐204 binding site, the alternation of the luciferase activity was abolished (Figure 3E). The data indicate that PCAT6 interacts with miR‐204 through direct binding.

### 3.4 MiR‐204 reverses the effect of PCAT6 on CRC cell chemoresistance through HMGA2/PI3K signaling

**Figure 4:**
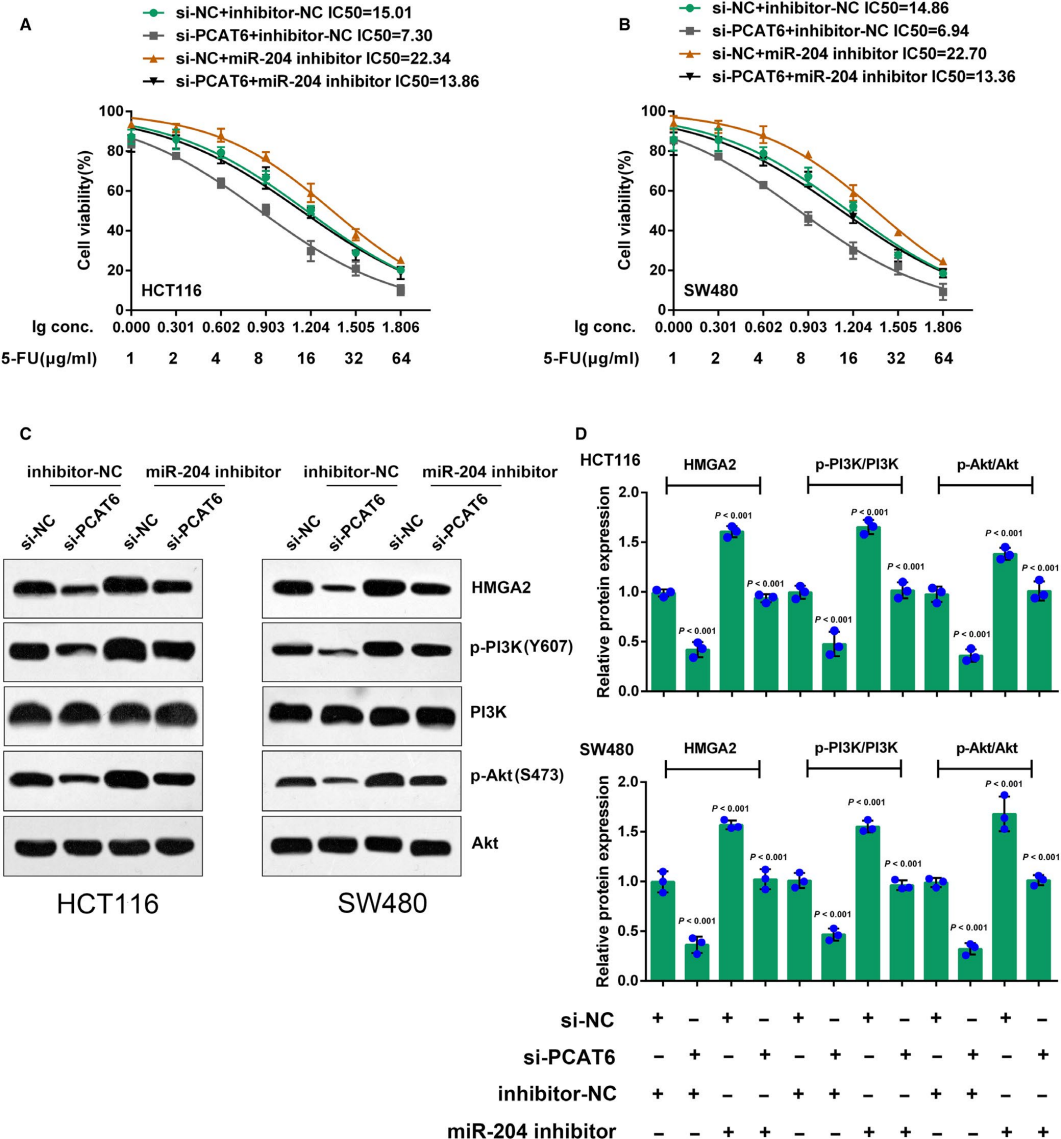
MiR‐204 reverses the effect of PCAT6 on CRC cell chemoresistance through HMGA2/PI3K signaling. (A‐B) HCT116 and SW480 cells were cotransfected with si‐PCAT6 and miR‐204 inhibitor, and were treated with a series of doses of 5‐FU (1, 2, 4, 8, 16, 32, 64 μg/mL) for 24 h; the cell viability was determined using MTT assays. The data were processed as described. (C‐E) HCT116 and SW480 cells were cotransfected with the si‐PCAT6 and miR‐204 inhibitor; the protein levels of HMGA2, p‐PI3K, PI3K, p‐Akt, and Akt were determined using Western blot assays; the ratio of p‐PI3K/PI3K and p‐Akt/Akt was shown. The data are presented as mean ± SD of three independent experiments

After confirming that miR‐204 is a direct target of PCAT6, we further investigated the function and mechanism of PCAT6/miR‐204 interaction in CRC cell chemoresistance. HCT116 and SW480 cells were cotransfected with si‐PCAT6 and miR‐204 inhibitor, exposed to different concentrations of 5‐FU (1, 2, 4, 8, 16, 32, and 64 μg/mL) for 24 hours, and then subjected to MTT assays for cell viability. 5‐FU suppressed the cell viability in a dose‐dependent manner; PCAT6 knockdown reduced whereas miR‐204 inhibition promoted the IC50 values for both HCT116 and SW480 cells; the effect of PCAT6 knockdown was partially reversed by miR‐204 inhibition (Figure 4A,B). In other words, PCAT6 knockdown sensitized CRC cells to 5‐FU treatment, while miR‐204 inhibition enhanced the chemoresistance of CRC cells.

Furthermore, we monitored HMGA2 signaling‐related proteins in response to the combined effect of PCAT6 knockdown and miR‐204 inhibition. As shown by Immunoblotting, the protein levels of HMGA2, p‐PI3K, and p‐Akt were dramatically reduced by PCAT6 knockdown while increased by miR‐204 inhibition without apparent changes in total PI3K and Akt protein expression (Figure 4C‐E); the suppressive effects of PCAT6 knocking down on these proteins were partially attenuated by miR‐204 inhibition (Figure 4C‐E). As shown in Figure 4D,E, the ratio of p‐PI3K/PI3K and p‐Akt/Akt was reduced by PCAT6 knockdown while increased by miR‐204 inhibition; the effect of PCAT6 was partially abolished by miR‐204 inhibition, indicating PCAT6 affects CRC cell chemoresistance through miR‐204; HMGA2/PI3K signaling is involved in this regulatory process.

### 3.5 The expression and correlation of miR‐204, HMGA2, and PCAT6 in CRC tissues

**Figure 5:**
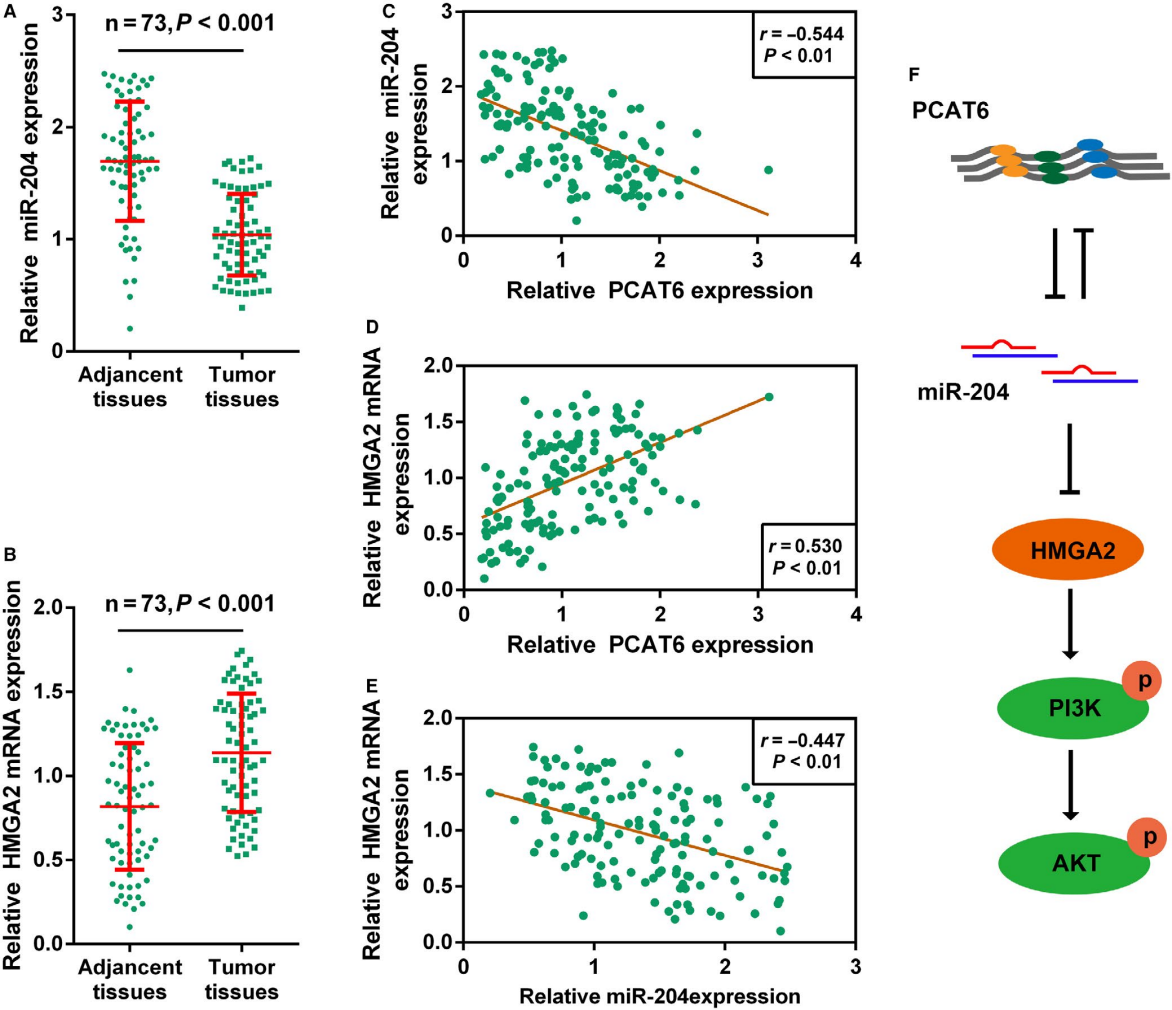
The expression and correlation of miR‐204, HMGA2, and PCAT6 in CRC tissues. (A‐B) The expression of miR‐204 and HMGA2 in 73 paired CRC and adjacent normal tissues were determined using real‐time PCR assays. (C‐E) The correlation between miR‐204 and HMGA2, between miR‐204 and PCAT6, and between HMGA2 and PCAT6 was analyzed using Spearman's rank correlation analysis. (F) A mechanism diagram showing PCAT6 interacts with miR‐204, thereby affecting the chemoresistance of CRC cell to 5‐FU‐based treatment through HMGA2/PI3K signaling pathway

To further confirm the above findings, miR‐204 and HMGA2 expression in CRC and non‐tumor tissue samples were examined by real‐time PCR assays. MiR‐204 expression was strongly downregulated while HMGA2 mRNA expression was increased in CRC tissue samples, compared that in adjacent non‐tumor tissues (Figure 5A,B), indicating that the expression levels of miR‐204 and HMGA2 were indeed dysregulated in CRC. A negative correlation between miR‐204 and PCAT6, between miR‐204 and HMGA2, and a positive correlation between PCAT6 and HMGA2 was observed (Figure 5C‐E). As shown in Figure 5F, PCAT6 interacts with miR‐204 to inhibit its expression, thus promoting the activation of HMGA2/PI3K signaling and affecting the chemoresistance of CRC cells, indicating that suppressing PCAT6 expression, thereby rescuing miR‐204 expression, ultimately improving the chemo‐sensitivity of CRC cells to 5‐FU appears to be a promising adjuvant treatment to enhance the efficacy of 5‐FU‐based therapy.

## 4 DISCUSSION

Here, we elaborated a lncRNA, namely PCAT6, is over‐expressed in CRC tissue samples; a higher PCAT6 expression is correlated with a poorer prognosis in patients with CRC. Moreover, PCAT6 directly binds to miR‐204 to inhibit its expression, and enhance CRC chemoresistance to 5‐FU‐based therapy through HMGA2. In CRC tissues, the expression levels of PCAT6, miR‐204, and HMGA2 were all dysregulated, indicating that modulating the above regulatory axis may ameliorate the chemoresistance of CRC to 5‐FU‐based chemotherapy.

According to previous studies, approximately up to 18% of ncRNAs participate in pathogenic processes, including carcinogenesis,^35^ indicating that lncRNA, a significant part of ncRNAs, might make contributions to cancer initiation and progression. LncRNA dysregulation commonly occurs in many cancers, affecting cancer progression from almost every aspect of carcinogenesis, including the resistance of cancer cells to chemo‐ and radiotherapy. LncRNA HOTAIR can enhance the chemoresistance of lung cancer and glioma cells by blocking p21.^36^, ^37^ LncRNA UCA1 reduces the sensitivity of CRC cells to 5‐FU‐based chemotherapy through hindering miR‐204‐5p.^38^ Low XIST expression is correlated with drug response in Patient‐Derived Xenografts (PDXs) accompanied by a significant reduction of the breast cancer stem cell population.^39^ PCAT6 is one member of Prostate Cancer Associated Transcripts (PCATs), a series of 121 lncRNA which were first identified in prostate cancer by using computational bioinformatics means to describe the annotated and unannotated transcripts in prostate cancer.^40^ The expression patterns of PCATs could help identify samples of benign, localized, and metastatic cancer.^40^ As we mentioned, abnormal lncRNA PCAT6 expression is observed in many cancers.^28^, ^29^ In Wan et al's study, PCAT6 was considered as a potential diagnostic and prognostic biomarker in non‐small cell lung cancer.^41^ PCAT6 may indirectly regulate c‐Myc and p53 expression in lung cancer which may contribute to the modulation of lung cancer cells proliferation and invasion.^42^ In the present study, we also observed higher PCAT6 expression in CRC tissues. Higher PCAT6 expression is associated with shorter overall survival, as well as poorer clinical features in patients with CRC. In CRC cell lines, the PCAT6 expression is also upregulated. After the PCAT6 knockdown, cancer cell proliferation was remarkably suppressed; also, PCAT6 knockdown enhanced CRC sensitivity to 5‐FU treatment. What is the downstream mechanism of PCAT6 affecting CRC cell proliferation and chemoresistance to 5‐FU?

In our previous study, we demonstrated a miR‐204/HMGA2 axis which can modulate CRC cell chemoresistance to 5‐FU‐based chemotherapy.^5^ Herein, we also validated that PCAT6 knockdown could reduce HMGA2 protein level. To further investigate whether PCAT6 affects CRC cell chemoresistance through the miR‐204/HMGA2 axis, we monitored miR‐204 expression in PCAT6 knocked‐down cells. On the contrary of HMGA2, miR‐204 expression was significantly upregulated by the PCAT6 knockdown. Moreover, PCAT6 also showed to be negatively regulated by miR‐204, suggesting that PCAT6 may interact with miR‐204 to inhibit its expression. In order to validate this interaction, luciferase reporter gene assays were performed; as the results showed, PCAT6 could directly bind to miR‐204 to inhibit its expression. Based on our previous study, abnormal downregulation of miR‐204 expression in CRC contributes to the chemoresistance to 5‐FU; whether miR‐204 is involved in PCAT6 regulation of CRC cell chemoresistance to 5‐FU remains unclear.

In addition to our previous study, there are many other references regarding miRNAs as essential regulators of cancer cell proliferation and chemoresistance. In epithelial ovarian cancer, IL‐6 receptor (IL‐6R), which could activate STAT3 in IL‐6‐dependent manner, could be targeted by miR‐204. Moreover, the activity of IL‐6R/STAT3/miR‐204 feedback loop is correlated with chemo‐sensitivity of epithelial ovarian cancer.^43^ In another research on tumor‐related macrophages in CRC, the maladjusted miR‐155‐5p/C/EBPβ/IL‐6 signaling could induce chemoresistance in CRC cells by regulating the IL‐6R/STAT3/miR‐204‐5p axis.^44^ Through direct binding to ZEB1, miR‐204 modulates chemo‐sensitivity and apoptosis of prostate cancer cells.^45^ We have revealed that PCAT6 directly binds to miR‐204 to inhibit its expression; herein, we also assessed the combined effect of PCAT6 and miR‐204 on the chemoresistance of CRC cells to 5‐FU. PCAT6 knockdown attenuated, whereas miR‐204 inhibition enhanced CRC chemoresistance to 5‐FU; miR‐204 inhibition could partially reverse the effect of PCAT6 knockdown. Moreover, HMGA2 signaling‐related factors could also be affected by co‐transfection of si‐PCAT6 and miR‐204 inhibitor. PCAT6 knockdown reduced the protein level of HMGA2 and the ratio of p‐PI3K/PI3K and p‐Akt/Akt, indicating that PCAT6 knockdown inhibited the activity of HMGA2/PI3K signaling. On the contrary, miR‐204 inhibition promoted the activity of HMGA2/PI3K signaling by increasing the protein level of HMGA2, as well as the phosphorylation of PI3K and Akt. In the meantime, miR‐204 partially attenuated the cell effect of PCAT6 knockdown on HMGA2/PI3K signaling, indicating that PCAT6 exerts its function through the miR‐204/HMGA2 axis.

To further validate the above findings, we monitored miR‐204 and HMGA2 mRNA expression in CRC and non‐tumor tissue samples. Consistent with our previous study, miR‐204 expression was downregulated, whereas HMGA2 mRNA expression was upregulated in CRC tissues. Moreover, miR‐204 expression was inversely correlated with HMGA2 and PCAT6 expression, respectively; HMGA2 expression was positively associated with PCAT6 expression. The data indicate that abnormal PCAT6 overexpression inhibits miR‐204 expression in CRC, thereby promoting HMGA2/PI3K signaling activity, ultimately enhancing CRC chemoresistance to 5‐FU.

Taken together, we demonstrated that PCAT6 could modulate CRC chemoresistance to 5‐FU‐based chemotherapy through miR‐204/HMGA2/PI3K; PCAT6 represents a promising target for dealing with CRC chemoresistance.

## References

[1] Ferlay J , Shin HR , Bray F , Forman D , Mathers C , Parkin DM . Estimates of worldwide burden of cancer in 2008: GLOBOCAN 2008. Int J Cancer. 2010;127:2893‐2917. 21351269 10.1002/ijc.25516

[2] Kemeny N . Colorectal cancer–an undertreated disease. Anticancer Drugs. 1996;7:623‐629. 8913429 10.1097/00001813-199608000-00001

[3] Marschner N , Arnold D , Engel E , et al. Oxaliplatin‐based first‐line chemotherapy is associated with improved overall survival compared to first‐line treatment with irinotecan‐based chemotherapy in patients with metastatic colorectal cancer – Results from a prospective cohort study. Clin Epidemiol. 2015;7:295‐303. 25945067 10.2147/CLEP.S73857 PMC4408959

[4] Hind D , Tappenden P , Tumur I , Eggington S , Sutcliffe P , Ryan A . The use of irinotecan, oxaliplatin and raltitrexed for the treatment of advanced colorectal cancer: systematic review and economic evaluation. Health Technol Assess. 2008;12: iii‐ix:xi‐162. 10.3310/hta12150 18462574

[5] Wu H , Liang Y , Shen L . MicroRNA‐204 modulates colorectal cancer cell sensitivity in response to 5‐fluorouracil‐based treatment by targeting high mobility group protein A2. Biol Open. 2016;5:563‐570. 27095441 10.1242/bio.015008 PMC4874347

[6] Prensner JR , Chinnaiyan AM . The emergence of lncRNAs in cancer biology. Cancer Discov. 2011;1:391‐407. 22096659 10.1158/2159-8290.CD-11-0209 PMC3215093

[7] Spizzo R , Almeida MI , Colombatti A , Calin GA . Long non‐coding RNAs and cancer: a new frontier of translational research? Oncogene. 2012;31:4577‐4587. 22266873 10.1038/onc.2011.621 PMC3433647

[8] Ponting CP , Oliver PL , Reik W . Evolution and functions of long noncoding RNAs. Cell. 2009;136:629‐641. 19239885 10.1016/j.cell.2009.02.006

[9] Mercer TR , Dinger ME , Mattick JS . Long non‐coding RNAs: insights into functions. Nat Rev Genet. 2009;10:155‐159. 19188922 10.1038/nrg2521

[10] Hung CL , Wang LY , Yu YL , et al. A long noncoding RNA connects c‐Myc to tumor metabolism. Proc Natl Acad Sci USA. 2014;111:18697‐18702. 25512540 10.1073/pnas.1415669112 PMC4284533

[11] Guttman M , Donaghey J , Carey BW , et al. lincRNAs act in the circuitry controlling pluripotency and differentiation. Nature. 2011;477:295‐300. 21874018 10.1038/nature10398 PMC3175327

[12] Li Z , Shen J , Chan MT , Wu WK . TUG1: a pivotal oncogenic long non‐coding RNA of human cancers. Cell Prolif. 2016;49:471‐475. 27339553 10.1111/cpr.12269 PMC6496395

[13] Lian Y , Cai Z , Gong H , Xue S , Wu D , Wang K . HOTTIP: a critical oncogenic long non‐coding RNA in human cancers. Mol Biosyst. 2016;12:3247‐3253. 27546609 10.1039/c6mb00475j

[14] Ma Y , Lu Y , Lu B . MicroRNA and long non‐coding RNA in ovarian carcinoma: translational insights and potential clinical applications. Cancer Invest. 2016;34:465‐476. 27673409 10.1080/07357907.2016.1227446

[15] Xin Y , Li Z , Zheng H , Chan M . Ka Kei Wu W. CCAT2: A novel oncogenic long non‐coding RNA in human cancers. Cell Prolif. 2017;50. 10.1111/cpr.12342 PMC6529071 28244168

[16] Rinn JL , Chang HY . Genome regulation by long noncoding RNAs. Annu Rev Biochem. 2012;81:145‐166. 22663078 10.1146/annurev-biochem-051410-092902 PMC3858397

[17] Tsai MC , Manor O , Wan Y , et al. Long noncoding RNA as modular scaffold of histone modification complexes. Science. 2010;329:689‐693. 20616235 10.1126/science.1192002 PMC2967777

[18] Tripathi V , Ellis JD , Shen Z , et al. The nuclear‐retained noncoding RNA MALAT1 regulates alternative splicing by modulating SR splicing factor phosphorylation. Mol Cell. 2010;39:925‐938. 20797886 10.1016/j.molcel.2010.08.011 PMC4158944

[19] Kretz M , Siprashvili Z , Chu C , et al. Control of somatic tissue differentiation by the long non‐coding RNA TINCR. Nature. 2013;493:231‐235. 23201690 10.1038/nature11661 PMC3674581

[20] Karreth FA , Tay Y , Perna D , et al. In vivo identification of tumor‐ suppressive PTEN ceRNAs in an oncogenic BRAF‐induced mouse model of melanoma. Cell. 2011;147:382‐395. 22000016 10.1016/j.cell.2011.09.032 PMC3236086

[21] Hansen TB , Jensen TI , Clausen BH , et al. Natural RNA circles function as efficient microRNA sponges. Nature. 2013;495:384‐388. 23446346 10.1038/nature11993

[22] Gong C , Maquat LE . lncRNAs transactivate STAU1‐mediated mRNA decay by duplexing with 3' UTRs via Alu elements. Nature. 2011;470:284‐288. 21307942 10.1038/nature09701 PMC3073508

[23] Lalevee S , Feil R . Long noncoding RNAs in human disease: emerging mechanisms and therapeutic strategies. Epigenomics. 2015;7:877‐879. 26418705 10.2217/epi.15.55

[24] Fang Q , Chen X , Zhi X . Long non‐coding RNA (LncRNA) urothelial carcinoma associated 1 (UCA1) increases multi‐drug resistance of gastric cancer via downregulating miR‐27b. Med Sci Monit. 2016;22:3506‐3513. 27694794 10.12659/MSM.900688 PMC5051552

[25] Li Y , Jiang B , Zhu H , et al. Inhibition of long non‐coding RNA ROR reverses resistance to Tamoxifen by inducing autophagy in breast cancer. Tumour Biol. 2017;39:1010428317705790. 28635401 10.1177/1010428317705790

[26] Ragusa M , Barbagallo C , Statello L , et al. Non‐coding landscapes of colorectal cancer. World J Gastroentero. 2015;21:11709‐11739. 10.3748/wjg.v21.i41.11709 PMC4631972 26556998

[27] Orom UA , Derrien T , Beringer M , et al. Long noncoding RNAs with enhancer‐like function in human cells. Cell. 2010;143:46‐58. 20887892 10.1016/j.cell.2010.09.001 PMC4108080

[28] Du Z , Fei T , Verhaak RG , et al. Integrative genomic analyses reveal clinically relevant long noncoding RNAs in human cancer. Nat Struct Mol Biol. 2013;20:908‐913. 23728290 10.1038/nsmb.2591 PMC3702647

[29] Yang J , Lin J , Liu T , et al. Analysis of lncRNA expression profiles in non‐small cell lung cancers (NSCLC) and their clinical subtypes. Lung Cancer. 2014;85:110‐115. 24906504 10.1016/j.lungcan.2014.05.011

[30] Saunders NA . An Introduction to real‐time PCR. Wwwhorizon Presscom/pcr. 2014;98:444‐447.

[31] Liao Y , Shen L , Zhao H , et al. LncRNA CASC2 interacts with miR‐181a to modulate glioma growth and resistance to TMZ through PTEN pathway. J Cell Biochem. 2017;118:1889‐1899. 28121023 10.1002/jcb.25910

[32] Collins LJ . The RNA infrastructure: an introduction to ncRNA networks. Adv Exp Med Biol. 2011;722:1‐19. 21915779 10.1007/978-1-4614-0332-6_1

[33] Xu J , Li CX , Li YS , et al. MiRNA‐miRNA synergistic network: construction via co‐regulating functional modules and disease miRNA topological features. Nucleic Acids Res. 2011;39:825‐836. 20929877 10.1093/nar/gkq832 PMC3035454

[34] Jeggari A , Marks DS , Larsson E . miRcode: a map of putative microRNA target sites in the long non‐coding transcriptome. Bioinformatics. 2012;28:2062‐2063. 22718787 10.1093/bioinformatics/bts344 PMC3400968

[35] Khachane AN , Harrison PM . Mining mammalian transcript data for functional long non‐coding RNAs. PLoS One. 2010;5:e10316. 20428234 10.1371/journal.pone.0010316 PMC2859052

[36] Liu Z , Sun M , Lu K , et al. The long noncoding RNA HOTAIR contributes to cisplatin resistance of human lung adenocarcinoma cells via downregualtion of p21(WAF1/CIP1) expression. PLoS One. 2013;8:e77293. 24155936 10.1371/journal.pone.0077293 PMC3796503

[37] Jing L , Yuan W , Ruofan D , Jinjin Y , Haifeng Q . HOTAIR enhanced aggressive biological behaviors and induced radio‐resistance via inhibiting p21 in cervical cancer. Tumour Biol. 2015;36:3611‐3619. 25547435 10.1007/s13277-014-2998-2

[38] Bian Z , Jin L , Zhang J , et al. LncRNA‐UCA1 enhances cell proliferation and 5‐fluorouracil resistance in colorectal cancer by inhibiting miR‐204‐5p. Sci Rep. 2016;6:23892. 27046651 10.1038/srep23892 PMC4820696

[39] Salvador MA , Wicinski J , Cabaud O , et al. The histone deacetylase inhibitor abexinostat induces cancer stem cells differentiation in breast cancer with low Xist expression. Clin Cancer Res. 2013;19:6520‐6531. 24141629 10.1158/1078-0432.CCR-13-0877

[40] Prensner JR , Iyer MK , Balbin OA , et al. Transcriptome sequencing across a prostate cancer cohort identifies PCAT‐1, an unannotated lincRNA implicated in disease progression. Nat Biotechnol. 2011;29:742‐749. 21804560 10.1038/nbt.1914 PMC3152676

[41] Li W , Lin Z , Kai F , Wang JJ . Diagnostic significance of circulating long noncoding RNA PCAT6 in patients with non‐small cell lung cancer. Onco Targets Ther. 2017;10:5695. 29238201 10.2147/OTT.S149314 PMC5713690

[42] Wan L , Zhang L , Fan K , Cheng ZX , Sun QC , Wang JJ . Knockdown of long noncoding RNA PCAT6 inhibits proliferation and invasion in lung cancer cells. Oncol Res. 2016;24:161‐170. 27458097 10.3727/096504016X14618564639178 PMC7838661

[43] Zhu X , Shen H , Yin X , et al. IL‐6R/STAT3/miR‐204 feedback loop contributes to cisplatin resistance of epithelial ovarian cancer cells. Oncotarget. 2017;8:39154‐39166. 28388577 10.18632/oncotarget.16610 PMC5503602

[44] Yin Y , Yao S , Hu Y , et al. The immune‐microenvironment confers chemoresistance of colorectal cancer through macrophage‐derived IL‐6. Clin Cancer Res. 2017;23(23):7375‐7387. 28928161 10.1158/1078-0432.CCR-17-1283

[45] Wu G , Wang J , Chen G , Zhao X . microRNA‐204 modulates chemosensitivity and apoptosis of prostate cancer cells by targeting zinc‐finger E‐box‐binding homeobox 1 (ZEB1). Am J Transl Res. 2017;9:3599‐3610. 28861151 PMC5575174

