# Supplementary figures and images for: Long non‐coding RNA PCAT6 targets miR‐204 to modulate the chemoresistance of colorectal cancer cells to 5‐fluorouracil‐based treatment through HMGA2 signaling
Source: Cancer Med. 2019 Apr 1;8(5):2484–95. doi: 10.1002/cam4.1809 (PMC6536993; doi:10.1002/cam4.1809)

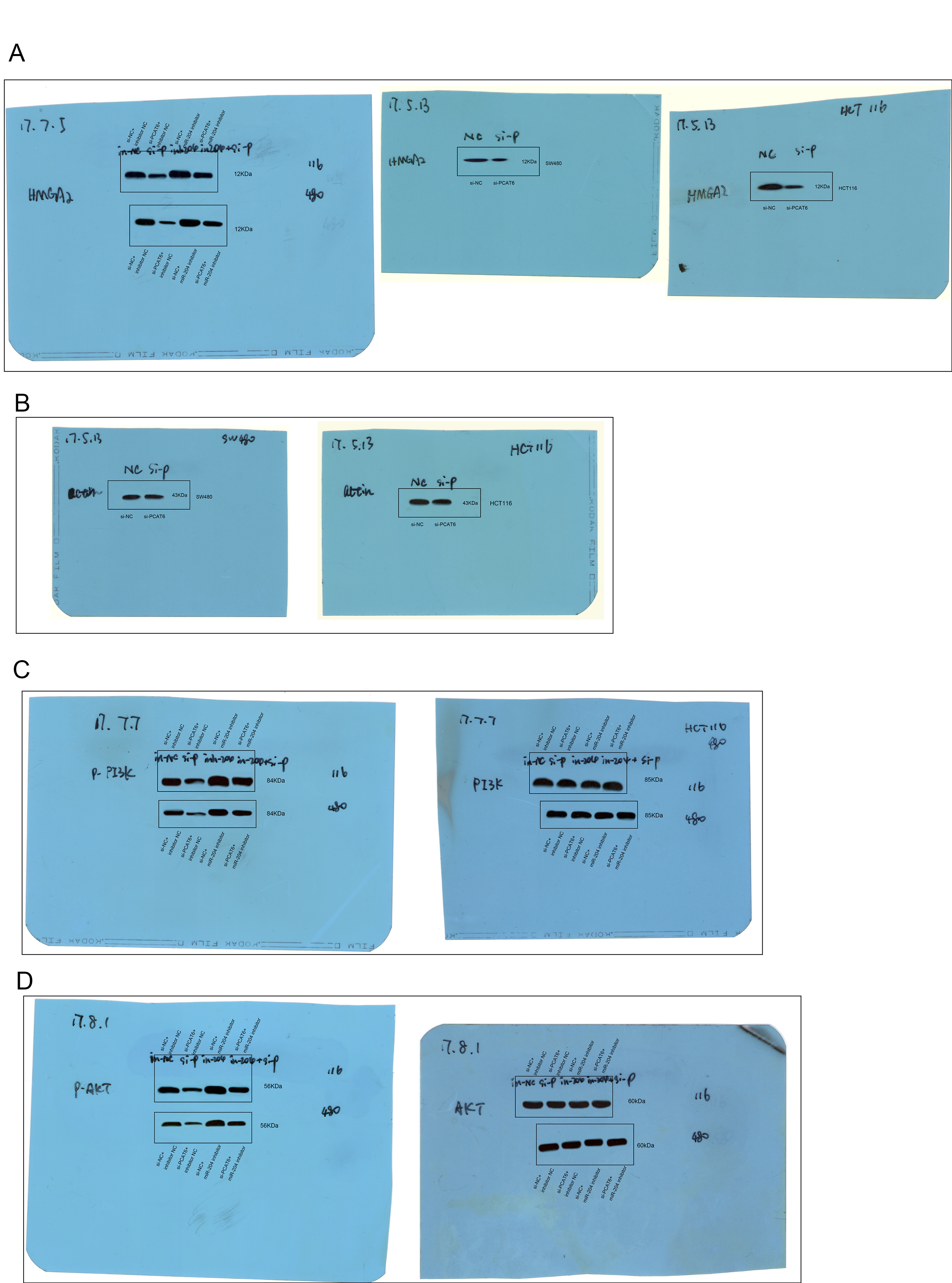

Supplement: Supplementary file 1 [file CAM4-8-2484-s001.tif]
