# Supplementary material for: Long non‐coding RNA PCAT6 targets miR‐204 to modulate the chemoresistance of colorectal cancer cells to 5‐fluorouracil‐based treatment through HMGA2 signaling
Source: Cancer Med. 2019 Apr 1;8(5):2484–95. doi: 10.1002/cam4.1809 (PMC6536993; doi:10.1002/cam4.1809)
Supplement: Supplementary file 2 [file CAM4-8-2484-s002.docx]

| PCAT6 | F: TTCCCTCGTCCTCTGCG | R: CAAGCGTTTGTGGGTTTCA |
| --- | --- | --- |
| MiR-204 | F: GCTTCCCTTTGTCATCCT | R: CAGTGCGTGTCGTGGA |
| HMGA2 | F: ACCCAGGGGAAGACCCAAA | R: CCTCTTGGCCGTTTTTCTCCA |
| GAPDH | F:GGAGCGAGATCCCTCCAAAAT | R:GGCTGTTGTCATACTTCTCATGG |
| U6 | F: CTCGCTTCGGCAGCACA | R: AACGCTTCACGAATTTGCGT |
| Si-NC | SENSE: TTCTCCGAACGTGTCACGT | ANTISENSE: ACGTGACACGTTCGGAGAA |
| Si-PCAT6 | SENSE : CUACAACGUCUUGUUACUATT | ANTISENSE : UAGUAACAAGACGUUGUAGTT |
| Mimics NC | SENSE:UUCUCCGAACGUGUCACGUTT | ANTISENSE: ACGUGACACGUUCGGAGAATT |
| Inhibitor NC | CAGUACUUUUGUGUAGUACAA |  |
| MiR-204 mimics | UUCCCUUUGUCAUCCUAUGC CU | GCAUAGGAUGACAAAGGGAAUU |
| MiR-204 inhibitor | AGGCAUAGGAUGACAAAGGGAA |  |
| Wt-PCAT6 | F:CCGCTCGAGTCTCCATCCTCATTCGGTC | R:ATAAGAATGCGGCCGCTTATAAGTGTTGTAATAGGGGGT |
| Mut-PCAT6 | F:GATGTCCCCTGTGAAACCCTGTTTCGCTTGCGATTTGGCC | R:GGCCAAATCGCAAGCGAAACAGGGTTTCACAGGGGACATC |

Table S1 The sequence of primers

|  |  |  |
| --- | --- | --- |
